# The Occurrence of Alzheimer’s Disease and Parkinson’s Disease in Individuals With Osteoporosis: A Longitudinal Follow-Up Study Using a National Health Screening Database in Korea

**DOI:** 10.3389/fnagi.2021.786337

**Published:** 2021-12-08

**Authors:** Mi Jung Kwon, Joo-Hee Kim, Ji Hee Kim, Seong Jin Cho, Eun Sook Nam, Hyo Geun Choi

**Affiliations:** ^1^Division of Neuropathology, Department of Pathology, Hallym University Sacred Heart Hospital, Hallym University College of Medicine, Anyang, South Korea; ^2^Division of Pulmonary, Allergy, and Critical Care Medicine, Department of Medicine, Hallym University Sacred Heart Hospital, Hallym University College of Medicine, Anyang, South Korea; ^3^Department of Neurosurgery, Hallym University Sacred Heart Hospital, Hallym University College of Medicine, Anyang, South Korea; ^4^Department of Pathology, Kangdong Sacred Heart Hospital, Hallym University College of Medicine, Seoul, South Korea; ^5^Department of Otorhinolaryngology-Head and Neck Surgery, Hallym University Sacred Heart Hospital, Hallym University College of Medicine, Anyang, South Korea

**Keywords:** osteoporosis, Alzheimer’s disease, Parkinson’s disease, longitudinal follow-up study, nationwide population-based cohort study

## Abstract

**Background**: Public health concerns regarding the potential link between osteoporosis and the increased occurrence of Alzheimer’s disease (AD) and Parkinson’s disease (PD) have been raised, but the results remain inconsistent and require further validation. Here, we investigated the long-term relationship of osteoporosis with the occurrence of AD/PD using data from a large-scale nationwide cohort.

**Methods**: This longitudinal follow-up study included 78,994 patients with osteoporosis and 78,994 controls from the Korean National Health Insurance Service-Health Screening Cohort database (2002–2015) who were matched using propensity score matching at a 1:1 ratio based on age, sex, income, and residential area. A Cox proportional hazard model was used to assess the association between osteoporosis and the occurrence of AD/PD after adjusting for multiple covariates.

**Results**: During the follow-up period, AD occurred in 5,856 patients with osteoporosis and 3,761 controls (incidence rates: 10.4 and 6.8 per 1,000 person-years, respectively), and PD occurred in 1,397 patients and 790 controls (incidence rates: 2.4 and 1.4 per 1,000 person-years, respectively). The incidences of AD and PD were significantly higher in the osteoporosis group than in the matched control group. After adjustment, the osteoporosis group exhibited 1.27-fold and 1.49-fold higher occurrences of AD (95% confidence interval (CI) = 1.22–1.32) and PD (95% CI = 1.36–1.63) than the controls, respectively. The results of subgroup analyses supported the increased occurrence of AD and PD in patients with osteoporosis, independent of income, residential area, obesity, smoking, alcohol consumption, hyperlipidemia, hypertension, or blood glucose level.

**Conclusion**: Our results indicate that the presence of osteoporosis may increase the likelihood of developing two common neurodegenerative diseases in adults aged ≥40 years.

## Introduction

Osteoporosis, Alzheimer’s disease (AD), and Parkinson’s disease (PD) are common chronic degenerative disorders that are strongly associated with advanced age and substantial morbidity and mortality (Park et al., 2011; Lee et al., 2021; Yoon et al., 2021). Considering the aging society in Korea, these diseases are becoming recognized as important public health concerns (Park et al., 2011; Lee et al., 2021; Yoon et al., 2021). AD is the most common neurodegenerative disease and the most frequent form of dementia, accounting for 75% of all cases (Lee et al., 2021). PD is the second most common neurodegenerative disease after AD and is characterized by four cardinal motor signs: tremor at rest, bradykinesia, rigidity, and postural instability (Gelb et al., 1999). These neurodegenerative diseases result from the gradual and progressive loss of neural cells because of abnormal deposits of β-amyloid and tau protein in patients with AD and α-synucleinopathy in dopaminergic neurons of the substantia nigra in patients with PD, leading to nervous system dysfunction (Gelb et al., 1999; Reitz and Mayeux, 2014). Osteoporosis is a common skeletal disorder that mainly affects the elderly and is characterized by reduced bone mineral density and bone quality, along with subsequent increases in bone fragility and fracture susceptibility (Park et al., 2011). Osteoporosis is estimated to affect more than 200 million people worldwide, and the prevalence is increasing (Reginster and Burlet, 2006). In Korea, 22.4% of people over 50 years of age have osteoporosis, and 59.5% of women and 23.8% of men are predicted to suffer one or more fragility fractures during their remaining lifetime (Park et al., 2011).

A parallel increase in neurodegenerative disorders such as AD and PD has been noted in countries where the prevalence of osteoporosis is steadily increasing (Roos, 2014). Over the last two decades, epidemiological studies have highlighted the potential link between osteoporosis and the risk of neurodegenerative disease (Yaffe et al., 1999; Tan et al., 2005; Zhou et al., 2011; Chang et al., 2014; Roos, 2014; Amouzougan et al., 2017; Kostev et al., 2018; Feng et al., 2021). Experimental evidence supports that molecular alterations and chemical exposure mediate the pathogenic changes associated with AD and PD in osteoporotic animal models (Li et al., 2013, 2014; Calabrese et al., 2016; Folke et al., 2019; Wang and Wang, 2020; Abdul-Latif et al., 2021; Jiang et al., 2021), and researchers assume they may share some central mechanisms. While the majority of epidemiological studies have focused on the effect of osteoporosis on the risk of AD or dementia (Yaffe et al., 1999; Tan et al., 2005; Zhou et al., 2011; Chang et al., 2014; Roos, 2014; Amouzougan et al., 2017; Kostev et al., 2018), data for the relation to PD risk are scarce (Feng et al., 2021). Fewer than 10 studies have been published on this topic since Yaffe et al. (1999) first reported that women over 65 years old with osteoporosis have a greater risk of cognitive deterioration. Afterward, a prospective cohort study including both women and men specifically showed that only women with a low bone mineral density are at 1.83-fold higher risk of developing AD (Tan et al., 2005). A recent large-scale retrospective study showed that osteoporosis was associated with a higher risk of dementia in women (1.2-fold) and men (1.3-fold) at the 20-year follow-up, warning that osteoporosis may develop in individuals of both sexes who are prone to dementia (Kostev et al., 2018). However, because that study (Kostev et al., 2018) did not specify the type of dementia, researchers have not clearly determined whether osteoporosis exerts a negative effect on the risk of AD in both sexes. The most recent study substantiated this claim, reporting an increased risk of PD in patients with osteoporosis over a relatively short 4-year follow-up period (Feng et al., 2021), which has yet to be reproduced in analyses of long-term follow-up data. Thus, the occurrence of osteoporosis-related adverse events is of significant importance in terms of public health strategies and socioeconomic burdens in aging societies (Park et al., 2011). Unfortunately, no study has yet established the incidence and risk of AD and PD, two representative neurodegenerative diseases, and their association with osteoporosis. Because osteoporosis, AD, and PD appear to share risk factors and reciprocal associations (Tysiewicz-Dudek et al., 2008; Zhang and Tian, 2014; Thulkar et al., 2016), a long-term follow-up study adjusting for possible mutual confounders is needed to validate the association of osteoporosis with the risks of AD/PD.

Thus, we hypothesized that osteoporosis might adversely affect the development of AD and PD. Here, using the nationwide Korean National Health Insurance Service database, we sought to investigate whether osteoporosis was associated with the increased occurrence of either incident AD or PD.

## Subjects and Methods

### Study Population and Participant Selection

The Ethics Committee of Hallym University (2019-10-023) approved this study. The requirement for written informed consent was waived by the Institutional Review Board. The present study used the Korean National Health Insurance Service-Health Screening Cohort (KNHIS-HSC) database, which contains longitudinal data. A detailed description of the KNHIS-HSC database has been provided elsewhere (Kim et al., 2019). The data files were deidentified by scrambling the identification codes of all beneficiaries, and information obtained from the database was entirely anonymous. The diagnostic codes used in the KNHIS-HSC database follow the International Classification of Diseases, Tenth Revision, Clinical Modification (ICD-10-CM).

We used a retrospective cohort study design to assess the effect of osteoporosis on people’s risk of subsequently developing AD or PD in two cohort groups: an osteoporosis group and a comparison group. The initial osteoporosis group was selected from 514,866 participants aged ≥40 years with 615,488,428 medical claim codes from a minimum of two clinic visits during the period ranging from 2002 through 2015 (*n* = 94,932). Osteoporosis was defined using the ICD-10 codes M80 (osteoporosis with pathological fracture), M81 (osteoporosis without pathological fracture), or M82 (osteoporosis in diseases classified elsewhere) and ≥2 historical bone density tests using X-ray or CT (Min et al., 2019; Kim et al., 2020a). We excluded any participants with osteoporosis who were diagnosed in 2002 (1-year wash-out period, *n* = 14,772), did not have records for total cholesterol levels (*n* = 13), blood pressure (*n* = 3), or body mass index (BMI, kg/m^2^, *n* = 4), or were diagnosed with AD/PD before their diagnosis of osteoporosis (*n* = 1,146). Because we excluded any participants diagnosed with osteoporosis in 2002, the final osteoporosis group included only patients diagnosed on or after January 1, 2003, and their earliest follow-up time in the study started on January 1, 2003.

The control group comprised participants who were not diagnosed with codes M80 to M82 from 2002 through 2015 (*n* = 357,193). Control participants were excluded if they had no records or information available since 2003 (*n* = 4).

**Figure 1:**
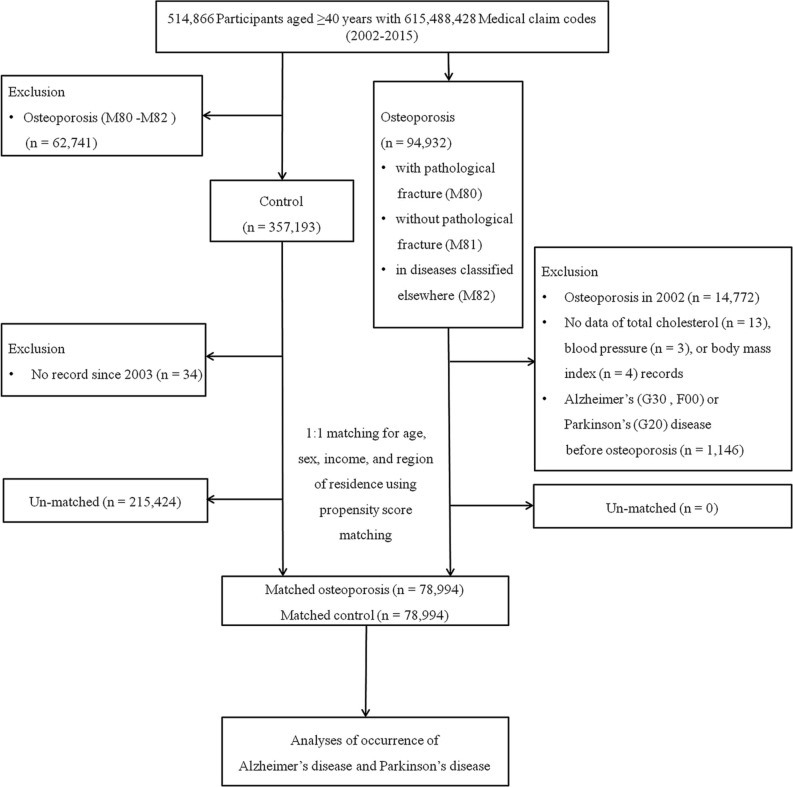
Schematic illustration of the participant selection process used in the present study. Of the total of 514,866 participants, 78,994 participants with osteoporosis were matched with 78,994 control participants for age, sex, income, and region of residence using propensity score matching.

Propensity score matching was performed based on age, sex, income, and region of residence to minimize the differences in the baseline demographic and clinical characteristics of the osteoporosis and control groups. The index date of each participant with osteoporosis was defined as the day when the ICD-10 codes for osteoporosis (M80–M82) were electronically assigned to patients in health insurance claims datasets. The index date of the control participants was defined as the index date of the matched participants with osteoporosis. Therefore, each matched patient with osteoporosis and control participant had the same index date. During the matching process, 215,424 control participants were excluded. Finally, 78,994 participants with osteoporosis were matched with 78,994 control participants at a 1:1 ratio (Figure 1).

Then, we searched for the occurrence of newly diagnosed AD or PD (newly assigned ICD-10 codes for AD or PD) in the osteoporosis and control groups between each individual’s index date and the end of 2015.

### Definition of Osteoporosis (Independent Variable)

Osteoporosis was defined using the ICD-10 codes M80-M82 (osteoporosis with pathological fracture, osteoporosis without pathological fracture, and osteoporosis in diseases classified elsewhere, respectively) ≥2 times with histories of bone density tests using X-ray or CT (Min et al., 2019; Kim et al., 2020a).

### Definitions of Alzheimer’s Disease and Parkinson’s Disease (Dependent Variables)

Alzheimer’s disease was defined using G30 (Alzheimer’s disease) or F00 (dementia in Alzheimer’s disease) ≥2 times, as described in our previous studies (Kim et al., 2018; Min et al., 2019).

Parkinson’s disease was defined using code G20 (Parkinson’s disease) ≥2 times, as described in our previous study (Choi et al., 2019).

### Covariates

The participants were divided into 10 age groups based on 5-year intervals and five income groups [class 1 (lowest income) to class 5 (highest income)]. The region of residence was stratified into urban and rural areas according to our previous study (Kim et al., 2019, 2021). Tobacco smoking, alcohol consumption, and obesity (BMI) were categorized in a manner similar to that described in our previous study (Kim et al., 2020b). Total cholesterol levels (mg/dL), systolic blood pressure (SBP, mmHg), diastolic blood pressure (DBP, mmHg), and fasting blood glucose levels (mg/dl) were measured. The Charlson Comorbidity Index (CCI) is widely applied to quantify the disease burden based on 17 comorbidities (acute myocardial infarction, congestive heart failure, peripheral vascular disease, cerebral vascular accident, dementia, pulmonary disease, connective tissue disorder, peptic ulcer, liver disease, diabetes, diabetes complications, paraplegia, renal disease, cancer, metastatic cancer, severe liver disease, and HIV). The CCI calculated for these comorbidities was summed as the continuous variable [0 (no comorbidities) to 29 (multiple comorbidities); Quan et al., 2011]. The CCI was calculated without AD or PD. We tried to use the clinical implications of the CCI to exclude comorbidities as possible risk factors for AD and PD because the increase in weight of these comorbidities may be related to an aging population and the increasing severity of disease in hospitalized patients (Quan et al., 2011). We adjusted the potential confounding factors of obesity, smoking, alcohol, DBP, SBP, fasting blood glucose, total cholesterol, and dyslipidemia (comprising metabolic syndrome), and CCI scores (comprising frailty and weakness) using overlap weighted models by multivariable conditional logistic regression.

### Statistical Analyses

Propensity score matching was conducted to reduce the effect of possible confounding factors and selection bias as a method to minimize the difference between the osteoporosis and control groups. Propensity scores were calculated by performing a multivariable logistic regression analysis of the baseline covariates, including age, sex, income, and area of residence (Yang and Dalton, 2012). Using this method, participants with osteoporosis were individually matched with control participants based on similar propensity score values. We assessed the balance of the matched data between the osteoporosis and control groups by calculating absolute standardized differences in covariates before and after matching to reduce bias. An absolute standardized difference of <0.20 indicates a good balance for a particular covariate (Austin, 2009). Imbalanced covariates with absolute standardized difference >0.20 after matching were further adjusted using the Cox proportional hazards model (Austin, 2009). During propensity score matching, we used a greedy, nearest-neighbor matching algorithm to form pairs of patients with osteoporosis and control participants, which avoids eliminating osteoporosis participants because the number of control participants was not sufficient to match with participants with osteoporosis due to the higher prevalence of osteoporosis in elderly females (Austin, 2009). Using this greedy matching option, the control participants whose propensity score was closest to that of the patients were paired (Austin, 2009).

The Kaplan-Meier analysis and log-rank test were used to analyze the cumulative probability of incident AD/PD in the osteoporosis group compared to the control group. A Cox proportional hazard model was used to analyze the hazard ratios (HRs) and 95% confidence intervals (CIs) for incident AD or PD among the participants with osteoporosis and compare them with those of the control participants. The proportional hazard assumptions were confirmed by constructing log-minus-log plots, and no violations of these assumptions were identified (Supplementary Figure 1). In this analysis, both crude and adjusted models were calculated. For incident AD, the model was adjusted for obesity, smoking status, alcohol consumption, total cholesterol level, SBP, DBP, fasting blood glucose level, CCI, and PD; for incident PD, the model was adjusted for the aforementioned variables along with AD instead of PD. Subgroup analyses were performed by stratifying the participants according to the covariates.

Two-tailed analyses were performed, and significance was defined as a *P*-value of less than 0.05. The Bonferroni correction was used for multiple comparisons. SAS version 9.4 (SAS Institute Inc., Cary, NC, USA) was used for all statistical analyses.

## Results

### Baseline Characteristics

This study included 78,994 people diagnosed with osteoporosis during the period from 2003 through 2015, of whom 69,856 (88.4%) were women, and the same number of control participants matched for age, sex, income, and region of residence were selected. The mean follow-up duration was 94.2 months (standard deviation 41.4 months) in the osteoporosis group and 91.1 months (standard deviation 42.5 months) in the control group. The baseline characteristics of both groups after propensity score matching are summarized in Table 1. A balance of covariates was achieved between the two groups, except for age. Because osteoporosis is a highly prevalent disease in older aged individuals, the age distribution of participants with osteoporosis was not evenly distributed. After the matching procedure, the standardized difference in age remained relatively high but decreased (from 1.2 to 0.59 for the standardized difference) compared to that before matching (Supplementary Table 1).

**Table 1:** General characteristics of the participants.

Characteristics	Total participants			
	Osteoporosis (*n* = 78,994)	Control (*n* = 78,994)	Standardized difference	*P*-value
Age (years, n, %)			0.59	<0.001*
40–44	892 (1.1)	892 (1.1)	
45–49	4,422 (5.6)	4,422 (5.6)	
50–54	10,002 (12.7)	10,002 (12.7)	
55–59	11,511 (14.6)	25,571 (32.4)	
60–64	13,458 (17.0)	15,268 (19.3)	
65–69	16,736 (21.2)	7,815 (9.9)	
70–74	12,761 (16.2)	6,556 (8.3)	
75–79	6,674 (8.5)	3,980 (5.0)	
80–84	2,207 (2.8)	2,232 (2.8)	
≥85	331 (0.4)	2,256 (2.9)	
Sex (n, %)			0.11	<0.001*
Male	9,138 (11.6)	12,075 (15.3)	
Female	69,856 (88.4)	66,919 (84.7)	
Income (n, %)			0.05	0.395
1 (lowest)	15,048 (19.1)	13,971 (17.7)	
2	11,332 (14.4)	11,778 (14.9)	
3	12,343 (15.6)	13,280 (16.8)	
4	15,869 (20.1)	16,052 (20.3)	
5 (highest)	24,402 (30.9)	23,913 (30.3)	
Region of residence (n, %)			0.02	<0.001*
Urban	30,828 (39.0)	29,955 (37.9)	
Rural	48,166 (61.0)	49,039 (62.1)	
Total cholesterol level (mg/dL, mean, SD)	204.2 (38.9)	204.7 (39.3)	0.01	0.012*
SBP (mmHg, mean, SD)	127.3 (17.9)	127.7 (18.6)	0.02	0.024*
DBP (mmHg, mean, SD)	77.9 (11.0)	78.5 (11.4)	0.05	<0.001*
Fasting blood glucose level (mg/dL, mean, SD)	97.8 (28.1)	100.1 (32.7)	0.08	<0.001*
Obesity^†^ (n, %)			0.11	<0.001*
Underweight	2,573 (3.3)	1,870 (2.4)	
Normal	29,444 (37.3)	27,190 (34.4)	
Overweight	20,665 (26.2)	20,734 (26.3)	
Obese I	23,879 (30.2)	25,806 (32.7)	
Obese II	2,433 (3.1)	3,394 (4.3)	
Smoking status (n, %)			0.08	<0.001*
Nonsmoker	72,716 (92.1)	71,005 (89.9)	
Past smoker	2,460 (3.1)	3,196 (4.1)	
Current smoker	3,818 (4.8)	4,793 (6.1)	
Alcohol consumption (n, %)			0.09	<0.001*
<1 time a week	70,097 (88.7)	67,667 (85.7)	
≥1 time a week	8,897 (11.3)	11,327 (14.3)	
CCI (score, n, %)			0.13	<0.001*
0	49,798 (63.0)	54,635 (69.2)	
1	13,667 (17.3)	10,741 (13.6)	
≥2	15,529 (19.7)	13,618 (17.2)	
Dementia (n, %)	5,856 (7.4)	3,761 (4.8)	0.11	<0.001*
Parkinson’s disease (n, %)	1,397 (1.8)	790 (1.0)	0.07	<0.001*

Abbreviations: CCI, Charlson comorbidity index; DBP, diastolic blood pressure; SBP, systolic blood pressure; SD, standard deviation. *Wilcoxon rank-sum test, significant at *P* < 0.05. ^†^Obesity (BMI, body mass index, kg/m^2^) was categorized as <18.5 (underweight), ≥18.5 to <23 (normal), ≥23 to <25 (overweight), ≥25 to <30 (obese I), and ≥30 (obese II).

### The Occurrence of AD

**Figure 2:**
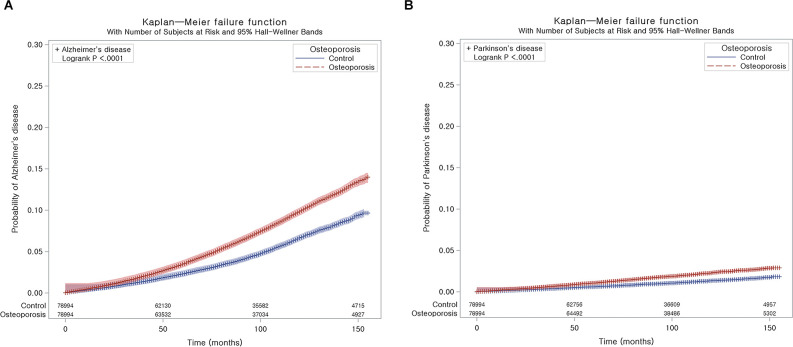
Kaplan-Meier probability of the incidence of neurodegenerative disease in osteoporosis and the control populations within 13 years of the index date: Alzheimer’s disease (AD) **(A)** and Parkinson’s disease (PD) **(B)**.

AD occurred in 5,856 (7.4%) patients with osteoporosis and 3,761 (4.8%) control participants. The incidence rates for AD were 10.4 and 6.8 per 1,000 person-years in the osteoporosis group and control group, respectively. The Kaplan-Meier analysis and log-rank test revealed a higher likelihood of developing AD in subjects who suffered from osteoporosis than in the control group (*P* < 0.0001; Figure 2A).

The Cox regression analysis adjusted for demographic data and medical comorbidities revealed that subjects with osteoporosis had a higher likelihood of developing AD than the controls (HR 1.27; 95% CI = 1.22–1.32; *P* < 0.001) at the 13-year follow-up (Table 2). The adjusted HR values for the effect of osteoporosis on developing AD in both the 5-year and 10-year follow-ups were also statistically significant [(1.29; 95% CI = 1.21–1.38; *P* < 0.001) and (1.27; 95% CI = 1.21–1.33; *P* < 0.001), respectively; Supplementary Table 2]. We gradually added covariates to calculate adjusted HRs in detail and to avoid potential interactions among the covariates (Supplementary Table 3). We observed prominent changes in the HR in Model 1 using age, sex, income, obesity, and region of residence as independent variables compared to other models.

**Table 2:** Hazard ratio (95% confidence interval) for Alzheimer’s disease in the osteoporosis and control groups with subgroup analyses stratified according to age and sex.

Characteristics	No. of individuals with AD/No. of participants	Follow-up duration, person-years	Incidence rate, per 1,000 person-years	Hazard ratios for AD			
				Crude	*P*-value	Adjusted^†^	*P*-value
Total participants (*n* = 157,988)
Osteoporosis	5,856/78,994 (7.4)	565,620	10.4	1.51 (1.45–1.58)	<0.001*	1.27 (1.22–1.32)	<0.001*
Control	3,761/78,994 (4.8)	551,763	6.8	1		1	
Age<60 years (*n* = 67,714)
Osteoporosis	293/26,827 (1.1)	220,194	16.1	1.37 (1.15–1.62)	<0.001*	1.44 (1.21–1.71)	<0.001*
Control	257/40,887 (0.6)	291,400	13.5	1		1
Age ≥60 years (*n* = 90,274)
Osteoporosis	5,563/52,167 (10.7)	345,426	16.1	1.24 (1.19–1.29)	<0.001*	1.22 (1.17–1.27)	<0.001*
Control	3,504/38,107 (9.2)	260,363	13.5	1		1
Males (*n* = 21,213)
Osteoporosis	712/9,138 (7.8)	48,608	14.6	0.85 (0.77–0.93)	0.001*	1.15 (1.04–1.28)	0.008*
Control	953/12,075 (7.9)	55,190	17.3	1		1
Females (*n* = 136,775)
Osteoporosis	5,144/69,856 (7.4)	517,012	9.9	1.76 (1.68–1.84)	<0.001*	1.28 (1.22–1.34)	<0.001*
Control	2,808/66,919 (4.2)	496,573	5.7	1		1	

Abbreviations: AD, Alzheimer’s disease. *Cox proportional hazard model, significant at *P* < 0.05 with the Bonferroni correction. ^†^Adjusted for age, sex, income, region of residence, total cholesterol level, SBP (systolic blood pressure), DBP (diastolic blood pressure), fasting blood glucose level, obesity, smoking, alcohol consumption, CCI (Charlson comorbidity index), and Parkinson’s disease.

**Figure 3:**
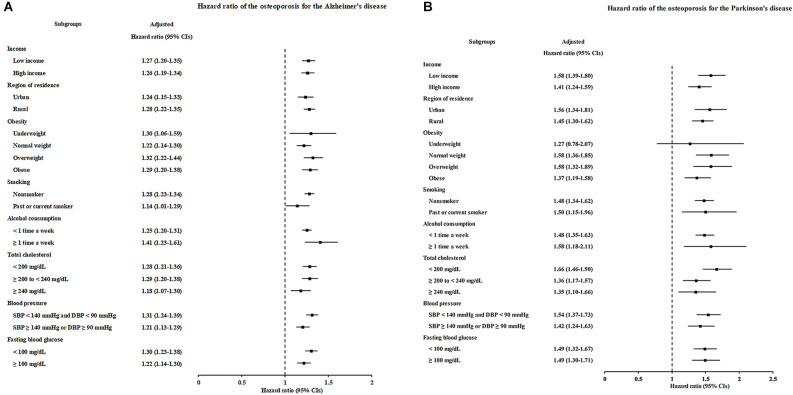
Forest plots depicting the association between osteoporosis and the subsequent risk of incident of Alzheimer’s disease (AD) **(A)** or Parkinson’s disease (PD) **(B)** in each subgroup.

As sex and age are associated with the occurrence of both osteoporosis and AD, we further stratified patients according to age and sex to determine the association between osteoporosis and the occurrence of AD. The occurrence of AD was significantly higher in the patients with osteoporosis who were either <60 years old or ≥60 years old [(1.44; 95% CI = 1.21–1.71; *P* < 0.001) and (1.22; 95% CI = 1.17–1.27; *P* < 0.001), respectively] and who were either males or females [(1.15; 95% CI = 1.04–1.28; *P* = 0.008) and (1.28; 95% CI = 1.22–1.34; *P* < 0.001), respectively]. Notably, the adjusted HRs of younger-aged participants and men were higher than the crude HRs. In other subgroup analyses, osteoporosis was significantly associated with elevated HRs of developing AD, regardless of income, region of residence, obesity, smoking status, alcohol consumption, total cholesterol level, hypertension, and blood glucose level (Figure 3A, Supplementary Table 4).

### The Occurrence of PD

During follow-up, PD occurred in 1,397 (1.8%) patients with osteoporosis and 790 (1.0%) control participants (Table 3). The incidence rates for PD were 2.4 and 1.4 per 1,000 person-years in the osteoporosis group and control group, respectively. As shown in Figure 2B, the incidence of PD was higher in patients with osteoporosis than in controls (*P* < 0.0001, log-rank test).

**Table 3:** Hazard ratio (95% confidence interval) for Parkinson’s disease in the osteoporosis and control groups with subgroup analyses stratified according to age and sex.

Characteristics	No. of individuals with AD/No. of participants	Follow-up duration, person-years	Incidence rate, per 1,000 person-years	Hazard ratios for AD			
				Crude	*P*-value	Adjusted^†^	*P*-value
Total participants (*n* = 157,988)	
Osteoporosis	1,397/78,994 (1.8)	577,566	2.4	1.72 (1.57–1.87)	<0.001*	1.49 (1.36–1.63)	<0.001*
Control	790/78,994 (1.0)	560,077	1.4	1		1
Age<60 years (*n* = 67,714)
Osteoporosis	129/26,827 (0.5)	220,598	0.6	1.50 (1.17–1.94)	0.002*	1.52 (1.17–1.98)	0.002*
Control	110/40,887 (0.3)	291,812	0.4	1		1
Age ≥60 years (*n* = 90,274)
Osteoporosis	1,268/52,167 (2.4)	356,968	3.6	1.41 (1.28–1.55)	<0.001*	1.40 (1.27–1.53)	<0.001*
Control	680/38,107 (1.8)	268,265	2.5	1		1
Males (*n* = 21,213)
Osteoporosis	210/9,138 (2.3)	49,754	4.2	1.31 (1.07–1.60)	0.008*	1.49 (1.21–1.83)	<0.001*
Control	184/12,075 (1.5)	56,728	3.2	1		1
Females (*n* = 136,775)
Osteoporosis	1,187/69,856 (1.7)	527,812	2.2	1.87 (1.69–2.06)	<0.001*	1.46 (1.33–1.62)	<0.001*
Control	606/66,919 (0.9)	503,349	1.2	1		1

Abbreviations: PD, Parkinson’s disease. *Cox proportional hazard model, significant at *P* < 0.05 with the Bonferroni correction. ^†^Adjusted for age, sex, income, region of residence, total cholesterol level, SBP, DBP, fasting blood glucose level, obesity, smoking, alcohol consumption, CCI score, and dementia.

A similar relationship was observed between osteoporosis and the occurrence of PD. Cox regression analyses revealed that the patients with osteoporosis had an increased likelihood of developing PD compared to the control group after adjusting for demographic data and medical comorbidities, including AD (HR 1.49; 95% CI = 1.36–1.63; *P* < 0.001), at the 13-year follow-up. The adjusted HR values for the effect of osteoporosis on incident PD at both the 5-year and 10-year follow-ups were also statistically significant [(1.50; 95% CI = 1.33–1.69; *P* < 0.001) and (1.50; 95% CI = 1.37–1.65; *P* < 0.001), respectively; Supplementary Table 2]. We also gradually added covariates to Models 1–4 to calculate adjusted HRs (Supplementary Table 5). A similar trend of a remarkable change in HR in Model 1 rather than other models was also observed, suggesting that the difference in age between the osteoporosis and comparison groups was considerable.

According to the subgroup analyses stratified by age and sex, the occurrence of PD was significantly increased in patients with osteoporosis who were either <60 years old or ≥60 years old [(1.52; 95% CI = 1.17–1.98; *P* = 0.002) and (1.40; 95% CI = 1.27–1.53; *P* < 0.001), respectively] and who were either males or females [(1.49; 95% CI = 1.21–1.83; *P* < 0.001) and (1.46; 95% CI = 1.33–1.62; *P* < 0.001), respectively]. However, a similar trend was also noted: the adjusted HRs for younger participants and men were higher than the crude HRs. The other subgroup analyses produced consistent findings: osteoporosis independently increased the occurrence of subsequent PD (Figure 3B, Supplementary Table 6).

We further examined the proportion of AD or PD diagnoses in the osteoporosis and control groups, respectively, according to the age distribution and sex using raw data (Supplementary Tables 7–10). As a result, the age distribution in older individuals over 65 and the female predominance of osteoporosis was relatively extreme.

## Discussion

Using nationwide large-scale cohort data, this longitudinal follow-up study revealed that patients with osteoporosis in the Korean population over 40 years old have a significantly increased incidence of both AD and PD across 5-year, 10-year, and 13-year follow-up intervals. This relationship between osteoporosis and the increased occurrence of AD and PD persisted even after adjusting for confounding factors. Our study highlights the substantial clinical importance of active treatment for osteoporosis to reduce the likelihood of AD and PD and prevent these neurodegenerative diseases in an aging society with an increasingly elderly population.

Our findings support the results of previous studies proposing an association between osteoporosis and the risk of AD or dementia (Yaffe et al., 1999; Tan et al., 2005; Zhou et al., 2011; Chang et al., 2014; Amouzougan et al., 2017; Kostev et al., 2018; Feng et al., 2021). Some studies have documented an association between osteoporosis and an increased AD risk only in women (Tan et al., 2005; Amouzougan et al., 2017). Other studies have indicated that both sexes with a low bone mineral density or osteoporosis are at risk of developing AD or dementia (Zhou et al., 2011; Kostev et al., 2018). Here, we found that both men and women with osteoporosis displayed a higher likelihood of developing AD than those in the matched control group, with a significantly increased HR observed independent of age, income, region of residence, obesity, smoking status, alcohol consumption, total cholesterol level, hypertension, and blood glucose level, suggesting that osteoporosis may be an early independent predictor of AD (Yaffe et al., 1999). In a Taiwanese longitudinal follow-up study, patients with osteoporosis exhibited a 1.47-fold higher risk of AD (95% CI = 1.10–1.98) than the control group (Chang et al., 2014). This finding is also consistent with our results that the osteoporosis group had a 1.27-fold higher risk of AD (95% CI = 1.22–1.32) than the matched control group (incidence rates: 10.4 and 6.8 per 1,000 person-years, respectively). However, some studies have reported results contradicting the association between AD or dementia and osteoporosis (Guo et al., 2012; Hsu et al., 2018), but these studies did not include a general population without osteoporosis as a control and had selection bias, as they evaluated either only older men aged 70–94 years or psychiatric patients.

Relevant studies on PD remain sparse, with only one recently published study identified (Feng et al., 2021). Similar to our study design and methodology using national health insurance claim data, this longitudinal follow-up study documented the association of osteoporosis with an increased risk of incident PD (Feng et al., 2021). The osteoporosis group had a 1.31-fold higher risk of PD (95% CI = 1.13–1.50) than the comparison group, with incidence rates of 2.46 and 1.94 per 1,000 person-years, respectively (Feng et al., 2021), which is highly comparable to our results indicating a 1.49-fold higher PD risk in the osteoporosis group than in the control group (95% CI = 1.36–1.63; incidence rates: 2.4 and 1.4 per 1,000 person-years, respectively). The previous study (Feng et al., 2021) showed that only women were affected when the patients were stratified by sex. In the present study, the relationships between osteoporosis and AD and PD risks were similar; the risk of patients with osteoporosis developing PD was significant and independent of income, region of residence, obesity, smoking, alcohol consumption, total cholesterol level, hypertension, blood glucose level, or AD. As a result, people over age 40 with osteoporosis were prone to AD and PD throughout 5-year, 10-year, and 13-year follow-up intervals compared to the matched general population. The effects of osteoporosis on incident AD and PD may be viewed *in toto* as independent risk factors for these neurodegenerative diseases throughout the follow-up period.

The pathophysiological associations between osteoporosis and AD and PD are unclear. AD, PD, and osteoporosis overlap in terms of some risk factors, including a lack of physical activity, smoking and alcohol consumption, comorbidities, obesity, heavy metal toxicity, inflammation, diabetes or hyperglycemia, and high cholesterol levels (Tysiewicz-Dudek et al., 2008; Zhang and Tian, 2014; Thulkar et al., 2016), which may contribute to systemic metabolic alterations accompanied by increased oxidative stress and persistent inflammatory conditions (Zhang and Tian, 2014), which might make susceptible elderly people more vulnerable to each key mechanism of AD, PD, and osteoporosis. Experimental studies have shown that the pathogenic proteins (β-amyloid, tau protein, and α-synuclein) related to AD and PD were detected in osteoporotic bone tissue, indicating that those proteins play a role in osteoporosis (Li et al., 2014; Calabrese et al., 2016). Research has identified several genetic or environmental factors that may contribute to the neurodegeneration associated with osteoporotic conditions. First, Wnt/β-catenin signaling has attracted attention because aberrant Wnt/β-catenin signaling has been reported to affect both osteoporosis and neurodegenerative diseases, including Parkinson’s and Alzheimer’s diseases (Li et al., 2013; Folke et al., 2019; Wang and Wang, 2020). Approximate percentages of patients with AD, PD, or osteoporosis who have a pathogenic variant of Wnt/β-catenin signaling have not yet been established. However, several studies assumed the proportion of pathogenic variants associated with Wnt/β-catenin signaling in these diseases (Nunes et al., 2007; Jia et al., 2019). One case-control study also showed a significantly lower incidence of AD (5% prevalence: 3/66) in elderly patients with bipolar disorder who were on lithium therapy, which is an agonist of Wnt signaling, compared to those without lithium therapy (33% prevalence: 16/48; Nunes et al., 2007). On the other hand, *LRRK2* mutations are closely related to canonical Wnt signaling and repress β-catenin, accounting for up to 40% of PD cases in some populations (Berwick and Harvey, 2012). Candidate osteoporosis genes commonly shared with AD and PD may include the *LRP5* and *LRP6* genes, which are partially affected by *LRRK2* mutations, in the canonical Wnt signaling pathway (van Meurs et al., 2006; Berwick and Harvey, 2012). Polymorphisms in the *LRP6* (Ilel062Val) gene and *LRP5* (Ala1330Val) gene have been shown to increase the fracture risk and comprise 10% of fractures in men (van Meurs et al., 2006). Women that harbored both alleles showed an insignificant trend of a 30% higher risk of fractures than other women (van Meurs et al., 2006).

Another plausible explanation might be that neurotoxicants and chronic inflammation induced by exposure to toxicants, such as copper, iron, cadmium, lead, aluminum, and arsenic, negatively affect bone mineral density (Uversky et al., 2001; Roos, 2014). Metals with neurotoxic properties have also been detected in the brain and cerebrospinal fluid from patients with neurodegenerative diseases, as they can cross the blood-brain barrier (Choi and Zheng, 2009). Inflammatory cytokines, including tumor necrosis factor-α, interleukin-1β, and interleukin-6, have been postulated to be involved in AD, PD, and osteoporosis (Zhang and Tian, 2014; Culibrk and Hahn, 2020). Third, oxidative stress and mitochondrial oxidative damage caused by aging may play a central role in these diseases because the risks of osteoporosis, AD, and PD increase significantly with age (Mattson et al., 1995; Yakunin et al., 2014; Domazetovic et al., 2017). The accumulation of hydrogen peroxide in neurons through β-amyloid in AD, inhibition of catalase activity by α-synuclein probably through hindering peroxisome proliferator-activated receptor *γ* (PPAR*γ*) transcriptional activity in PD, and decreases in GSH levels and intestinal absorption of antioxidants in elderly individuals with osteoporosis have been demonstrated to generate oxidative stress (Mattson et al., 1995; Yakunin et al., 2014; Domazetovic et al., 2017), which leads to cellular damage due to lipid oxidation, structural alteration of the membranes, and oxidation of proteins and nucleic acids (Domazetovic et al., 2017).

Identifying at-risk individuals by assessing known genetic susceptibility markers or potential risk factors might help precision medicine delay or prevent the development of clinical AD and PD (Titova and Chaudhuri, 2017). Our study has determined the clinical significance of osteoporosis as an independent risk factor to identify at-risk patients with susceptibility to AD and PD. Osteoporosis is also one of the comorbidities that may influence personalized and precision medicine strategies for elderly patients with AD or PD (Titova and Chaudhuri, 2017) because osteoporosis is highly prevalent with a median age of 75 years and a high risk of hip fracture (Wood and Walker, 2005; Tysiewicz-Dudek et al., 2008; Titova and Chaudhuri, 2017). Therefore, active, primary, and secondary prevention are required for all patients with osteoporosis, including vitamin D2, bisphosphonates, and physiotherapy, to reduce fear of falling and improve gait (Titova and Chaudhuri, 2017).

The strength of this study is based on the use of a representative, nationwide population data adjusted for socioeconomic status (e.g., area of residence and income) and lifestyle-related risk factors (e.g., total cholesterol level, alcohol, obesity, fasting blood glucose level, smoking, and blood pressure). Because the KNHIS-HSC data include all hospitals and clinics across the entire nation without exception, full medical histories were obtained for the follow-up period, increasing the generalizability of the study findings. To the best of our knowledge, this study represents the largest nationwide follow-up analysis of the association of osteoporosis with the risks of neurodegenerative diseases such as AD and PD.

Our research has limitations that must be addressed. Selection bias may be a weakness of this study. The control group was randomly matched (1:1) for age, sex, income, and region of residence to minimize selection bias, and the variables related to osteoporosis were adjusted for variables related to neurodegenerative disease to minimize confounding effects between osteoporosis and neurodegenerative disease. Nevertheless, because osteoporosis is highly prevalent in the female older age group, the large number of approximately 79,000 participants with osteoporosis could not be evenly matched to the corresponding control participants in each age group, which resulted in an imbalanced age distribution, even after applying propensity score matching. Although the standardized difference between men and women became acceptable after propensity score matching, the quite high female prevalence of osteoporosis was recognizable in the raw data comparison before the matching process. Therefore, the relationship between the increased occurrence of AD and PD in the age group <60 years old and men observed in our study must be interpreted with caution. Moreover, as this study was registered based on diagnosis codes and included only Korean subjects, unmeasured confounding effects and the possible risk factors could not be completely excluded. No information pertaining to bone mineral density, family history or genetic data for neurodegenerative disease or histories of prescriptions was lacking in this health insurance database, and thus the possibility of missing data was not considered.

## Conclusion

In summary, the results of this nationwide longitudinal follow-up study provide supporting evidence for the negative effect of osteoporosis on the development of two common neurodegenerative disorders.

## Data Availability Statement

The researcher cannot legally release the data. All data are available from the database of the National Health Insurance Sharing Service (NHISS) https://nhiss.nhis.or.kr/. NHISS provides access to all of these data to any researcher who promises to follow the research ethics requirements at some cost. If you want to access the data presented in this article, you can download them from the website after promising to follow the research ethics requirements.

## Author Contributions

HC: study conceptualization, funding acquisition, project administration, writing—review and editing. MK: investigation, writing—original draft, review and editing. J-HK: formal analysis and supervision. JK: formal analysis and methodology. EN: validation. SC: methodology and software. All authors contributed to the article and approved the submitted version.

## Conflict of Interest

The authors declare that the research was conducted in the absence of any commercial or financial relationships that could be construed as a potential conflict of interest.

## Publisher’s Note

All claims expressed in this article are solely those of the authors and do not necessarily represent those of their affiliated organizations, or those of the publisher, the editors and the reviewers. Any product that may be evaluated in this article, or claim that may be made by its manufacturer, is not guaranteed or endorsed by the publisher.
