# Supplementary material for: The Occurrence of Alzheimer’s Disease and Parkinson’s Disease in Individuals With Osteoporosis: A Longitudinal Follow-Up Study Using a National Health Screening Database in Korea
Source: Front Aging Neurosci. 2021 Dec 8;13:786337. doi: 10.3389/fnagi.2021.786337 (PMC8692765; doi:10.3389/fnagi.2021.786337)

**Supplementary Table** **1** General characteristics of participants for osteoporosis vs. raw data of control participants

| Characteristics | | Participants | | | |
| --- | --- | --- | --- | --- | --- |
|  |  | Osteoporosis (n = 78,994) | Without osteoporosis (n = 355,272) | Standardized  difference | *P* value |
| Age (years old, n, %) | |  |  | 1.22 | <0.001* |
|  | 40-44 | 892 (1.1) | 70,656 (19.9) |  |  |
|  | 45-49 | 4,422 (5.6) | 95,511 (26.9) |  |  |
|  | 50-54 | 10,002 (12.7) | 62,267 (17.5) |  |  |
|  | 55-59 | 11,511 (14.6) | 45,860 (12.9) |  |  |
|  | 60-64 | 13,458 (17.0) | 33,547 (9.4) |  |  |
|  | 65-69 | 16,736 (21.2) | 24,929 (7.0) |  |  |
|  | 70-74 | 12,761 (16.2) | 13,323 (3.8) |  |  |
|  | 75-79 | 6,674 (8.5) | 7,404 (2.1) |  |  |
|  | 80-84 | 2,207 (2.8) | 1,775 (0.5) |  |  |
|  | 85+ | 331 (0.4) | 0 (0.0) |  |  |
| Sex (n, %) | |  |  | 1.52 | <0.001* |
|  | Male | 9,138 (11.6) | 252,949 (71.2) |  |  |
|  | Female | 69,856 (88.4) | 102,323 (28.8) |  |  |
| Income (n, %) | |  |  | 0.16 | <0.001* |
|  | 1 (lowest) | 15,048 (19.1) | 50,028 (14.1) |  |  |
|  | 2 | 11,332 (14.4) | 45,901 (12.9) |  |  |
|  | 3 | 12,343 (15.6) | 55,463 (15.6) |  |  |
|  | 4 | 15,869 (20.1) | 75,811 (21.3) |  |  |
|  | 5 (highest) | 24,402 (30.9) | 128,069 (36.1) |  |  |
| Region of residence (n, %) | |  |  | 0.14 | <0.001* |
|  | Urban | 30,828 (39.0) | 162,719 (45.8) |  |  |
|  | Rural | 48,166 (61.0) | 192,553 (54.2) |  |  |
| Total cholesterol (mg/dL, mean, SD) | | 204.2 (38.9) | 199.0 (38.0) | 0.13 | <0.001* |
| SBP (mmHg, mean, SD) | | 127.3 (17.9) | 127.4 (18.0) | 0.00 | 0.913 |
| DBP (mmHg, mean, SD) | | 77.9 (11.0) | 80.2 (11.7) | 0.20 | <0.001* |
| Fasting blood glucose  (mg/dL, mean, SD) | | 97.8 (28.1) | 99.0 (35.1) | 0.04 | <0.001* |
| Obesity (n, %)‡ | |  |  | 0.10 | <0.001* |
|  | Underweight | 2,573 (3.3) | 7,589 (2.1) |  |  |
|  | Normal | 29,444 (37.3) | 122,971 (34.6) |  |  |
|  | Overweight | 20,665 (26.2) | 98,359 (27.7) |  |  |
|  | Obese I | 23,879 (30.2) | 116,603 (32.8) |  |  |
|  | Obese II | 2,433 (3.1) | 9,721 (2.7) |  |  |
| Smoking status (n, %) | |  |  | 0.83 | <0.001* |
|  | Nonsmoker | 72,716 (92.1) | 211,243 (59.5) |  |  |
|  | Past smoker | 2,460 (3.1) | 38,641 (10.9) |  |  |
|  | Current smoker | 3,818 (4.8) | 105,388 (29.7) |  |  |
| Alcohol consumption (n, %) | |  |  | 0.58 | <0.001* |
|  | < 1 time a week | 70,097 (88.7) | 232,525 (65.5) |  |  |
|  | ≥ 1 time a week | 8,897 (11.3) | 122,747 (34.6) |  |  |
| CCI score (score, n, %) | |  |  | 0.19 | <0.001* |
|  | 0 | 49,798 (63.0) | 254,080 (71.5) |  |  |
|  | 1 | 13,667 (17.3) | 43,833 (12.3) |  |  |
|  | ≥ 2 | 15,529 (19.7) | 57,359 (16.2) |  |  |
| Dementia (n, %) | | 5,856 (7.4) | 8,667 (2.4) | 0.23 | <0.001* |
| Parkinson’s disease (n, %) | | 1,397 (1.8) | 3,158 (0.9) | 0.08 | <0.001* |

Abbreviations: CCI, Charlson comorbidity index; DBP, diastolic blood pressure; SBP, systolic blood pressure; SD, standard deviation

* Wilcoxon rank-sum test. Significance at *P* < 0.05

† Obesity (BMI, body mass index, kg/m^2^) was categorized as < 18.5 (underweight), ≥ 18.5 to < 23 (normal), ≥ 23 to < 25 (overweight), ≥ 25 to < 30 (obese I), and ≥ 30 (obese II).

**Supplementary Table** **2** Hazard ratio (95% confidence interval) for Alzheimer’s disease and Parkinson’s disease in the osteoporosis and control groups of 5-year- and 10-year follow-up

| Characteristics | | Hazard ratio of 5-year follow-up | | | | Hazard ratio of 10-year follow-up | | | |
| --- | --- | --- | --- | --- | --- | --- | --- | --- | --- |
|  |  | Crude | *P*-value | Adjusted† | *P*-value | Crude | *P*-value | Adjusted† | *P*-value |
| Hazard ratio for Alzheimer’s disease | |  | |  |  |  |  |  |  |
|  | Osteoporosis | 1.51 (1.42-1.61) | <0.001* | 1.29 (1.21-1.38) | <0.001* | 1.53 (1.47-1.60) | <0.001* | 1.27 (1.21-1.33) | <0.001* |
|  | Control | 1 |  | 1 |  | 1 |  | 1 |  |
| Hazard ratio for Parkinson’s disease | |  | |  |  |  |  |  |  |
|  | Osteoporosis | 1.73 (1.54-1.95) | <0.001* | 1.50 (1.33-1.69) | <0.001* | 1.74 (1.59-1.91) | <0.001* | 1.50 (1.37-1.65) | <0.001* |
|  | Control | 1 |  | 1 |  | 1 |  | 1 |  |

* Cox proportional hazard model, Significance at *P* < 0.05 with Bonferroni correction

† Adjusted for age, sex, income, region, and obesity, smoking, alcohol consumption, total cholesterol, systolic blood pressure, diastolic blood pressure, and fasting blood glucose, Charlson comorbidity index (CCI) score, and Alzheimer’s disease (or Parkinson’s disease)

**Supplementary Table** **3** Hazard ratio (95% confidence interval) for Alzheimer's disease in the osteoporosis and control groups with subgroup analyses according to age and sex in multiple models

| Characteristics | | Hazard ratios for Alzheimer's disease | | | | | | | | | |
| --- | --- | --- | --- | --- | --- | --- | --- | --- | --- | --- | --- |
|  |  | Crude | *P*-value | Model 1† | *P*-value | Model 2‡ | *P*-value | Model 3§ | *P*-value | Model 4‖ | *P*-value |
| Total participants (n = 157,988) | |  | |  |  |  |  |  |  |  |  |
|  | Osteoporosis | 1.51 (1.45-1.58) | <0.001* | 1.30 (1.25-1.36) | <0.001* | 1.30 (1.25-1.36) | <0.001* | 1.32 (1.26-1.37) | <0.001* | 1.27 (1.22-1.32) | <0.001* |
|  | Control | 1 |  | 1 |  | 1 |  | 1 |  | 1 |  |
| Age < 60 years old (n = 67,714) | |  | |  |  |  |  |  |  |  |  |
|  | Osteoporosis | 1.37 (1.15-1.62) | <0.001* | 1.50 (1.27-1.78) | <0.001* | 1.51 (1.27-1.79) | <0.001* | 1.52 (1.28-1.80) | <0.001* | 1.44 (1.21-1.71) | <0.001* |
|  | Control | 1 |  | 1 |  | 1 |  | 1 |  | 1 |  |
| Age ≥ 60 years old (n = 90,274) | |  | |  |  |  |  |  |  |  |  |
|  | Osteoporosis | 1.24 (1.19-1.29) | <0.001* | 1.25 (1.19-1.30) | <0.001* | 1.25 (1.20-1.30) | <0.001* | 1.26 (1.21-1.31) | <0.001* | 1.22 (1.17-1.27) | <0.001* |
|  | Control | 1 |  | 1 |  | 1 |  | 1 |  | 1 |  |
| Males (n = 21,213) | |  | |  |  |  |  |  |  |  |  |
|  | Osteoporosis | 0.85 (0.77-0.93) | 0.001* | 1.21 (1.10-1.35) | <0.001* | 1.22 (1.10-1.35) | <0.001* | 1.23 (1.11-1.37) | <0.001* | 1.15 (1.04-1.28) | 0.008* |
|  | Control | 1 |  | 1 |  | 1 |  | 1 |  | 1 |  |
| Females (n = 136,775) | |  | |  |  |  |  |  |  |  |  |
|  | Osteoporosis | 1.76 (1.68-1.84) | <0.001* | 1.29 (1.23-1.35) | <0.001* | 1.30 (1.24-1.36) | <0.001* | 1.31 (1.25-1.37) | <0.001* | 1.28 (1.22-1.34) | <0.001* |
|  | Control | 1 |  | 1 |  | 1 |  | 1 |  | 1 |  |

Abbreviations: CCI, Charlson comorbidity index; DBP, diastolic blood pressure; SBP, systolic blood pressure

* Cox proportional hazard model, Significance at *P* <0.05 with Bonferroni correction

† Model 1 was adjusted for age, sex, income, region, and obesity

‡ Model 2 was adjusted for model 1 plus smoking, and alcohol consumption

§ Model 3 was adjusted for model 2 plus total cholesterol, SBP, DBP, and fasting blood glucose

‖ Model 4 was adjusted for model 3 plus CCI score, and Parkinson’s disease

**Supplementary Table** **4** Hazard ratio (95% confidence interval) for Alzheimer’s disease in the osteoporosis and control groups with subgroup analyses according to income, region of residence, obesity, smoking, alcohol consumption, total cholesterol, blood pressure, and fasting blood glucose

| Characteristics | | | No. of AD/ No. of participants | Follow-up duration, person-years | Incidence rate,  per 1,000  person-years | Hazard ratios for AD | | | |
| --- | --- | --- | --- | --- | --- | --- | --- | --- | --- |
|  |  |  |  |  |  | Crude | *P*-value | Adjusted† | *P*-value |
| Income | | | | | | | | | |
|  | Low income (n = 77,752) | | | | | | | | |
|  |  | Osteoporosis | 2,957/38,723 (7.6) | 276,711 | 10.7 | 1.53 (1.44-1.62) | <0.001* | 1.27 (1.20-1.35) | <0.001* |
|  |  | Control | 1,892/39,029 (4.9) | 271,727 | 7.0 | 1 |  | 1 |  |
|  | High income (n = 80,236) | | | | | | | | |
|  |  | Osteoporosis | 2,899/40,271 (7.2) | 288,909 | 10.0 | 1.50 (1.42-1.59) | <0.001* | 1.26 (1.19-1.34) | <0.001* |
|  |  | Control | 1,869/39,965 (4.7) | 280,036 | 6.7 | 1 |  | 1 |  |
| Region of residence | | | | | | | | | |
|  | Urban (n = 60,783) | | | | | | | | |
|  |  | Osteoporosis | 1,861/30,828 (6.0) | 225,236 | 8.3 | 1.42 (1.32-1.52) | <0.001* | 1.24 (1.15-1.33) | <0.001* |
|  |  | Control | 1,268/29,955 (4.2) | 216,131 | 5.9 | 1 |  | 1 |  |
|  | Rural (n = 97,205) | | | | | | | | |
|  |  | Osteoporosis | 3,995/48,166 (8.3) | 340,384 | 11.7 | 1.56 (1.49-1.64) | <0.001* | 1.28 (1.22-1.35) | <0.001* |
|  |  | Control | 2,493/49,039 (5.1) | 335,632 | 7.4 | 1 |  | 1 |  |
| Obesity | | | | | | | | | |
|  | Underweight (n = 4,443) | | | | | | | | |
|  |  | Osteoporosis | 251/2,573 (9.8) | 16,393 | 15.3 | 1.10 (0.90-1.35) | 0.334 | 1.30 (1.06-1.59) | 0.013* |
|  |  | Control | 156/1,870 (8.3) | 11,175 | 14.0 | 1 |  | 1 |  |
|  | Normal weight (n = 56,634) | | | | | | | | |
|  |  | Osteoporosis | 2,137/29,444 (7.3) | 207,747 | 10.3 | 1.35 (1.26-1.44) | <0.001* | 1.22 (1.14-1.30) | <0.001* |
|  |  | Control | 1,367/27,190 (5.0) | 180,522 | 7.6 | 1 |  | 1 |  |
|  | Overweight (n = 41,399) | | | | | | | | |
|  |  | Osteoporosis | 1,460/20,665 (7.1) | 149,352 | 9.8 | 1.58 (1.46-1.72) | <0.001* | 1.32 (1.22-1.44) | <0.001* |
|  |  | Control | 893/20,734 (4.3) | 145,657 | 6.1 | 1 |  | 1 |  |
|  | Obese (n = 55,512) | | | | | | | | |
|  |  | Osteoporosis | 2,008/26,312 (7.6) | 192,128 | 10.5 | 1.67 (1.56-1.79) | <0.001* | 1.29 (1.20-1.38) | <0.001* |
|  |  | Control | 1,345/29,200 (4.6) | 214,409 | 6.3 | 1 |  | 1 |  |
| Smoking | | | | | | | | | |
|  | Nonsmoker (n = 143,721) | | | | | | | | |
|  |  | Osteoporosis | 5,331/72,716 (7.3) | 529,106 | 10.1 | 1.61 (1.54-1.69) | <0.001* | 1.28 (1.23-1.34) | <0.001* |
|  |  | Control | 3,173/71,005 (4.5) | 509,627 | 6.2 | 1 |  | 1 |  |
|  | Past or current smoker (n = 14,267) | | | | | | | | |
|  |  | Osteoporosis | 525/6,278 (8.4) | 36,514 | 14.4 | 1.02 (0.91-1.15) | 0.692 | 1.14 (1.01-1.29) | 0.036 |
|  |  | Control | 588/7,989 (7.4) | 42,136 | 14.0 | 1 |  | 1 |  |
| Alcohol consumption | | | | | | | | | |
|  | < 1 time a week (n = 137,764) | | | | | | | | |
|  |  | Osteoporosis | 5,369/70,097 (7.7) | 518,451 | 10.4 | 1.53 (1.47-1.60) | <0.001* | 1.25 (1.20-1.31) | <0.001* |
|  |  | Control | 3,336/67,667 (4.9) | 494,658 | 6.7 | 1 |  | 1 |  |
|  | ≥ 1 time a week (n = 20,224) | | | | | | | | |
|  |  | Osteoporosis | 487/8,897 (5.5) | 47,169 | 10.3 | 1.37 (1.20-1.56) | <0.001* | 1.41 (1.23-1.61) | <0.001* |
|  |  | Control | 425/11,327 (3.8) | 57,105 | 7.4 | 1 |  | 1 |  |
| Total cholesterol | | | | | | | | | |
|  | < 200 mg/dL (n = 74,907) | | | | | | | | |
|  |  | Osteoporosis | 2,731/37,637 (7.3) | 264,251 | 10.3 | 1.45 (1.37-1.54) | <0.001* | 1.28 (1.21-1.36) | <0.001* |
|  |  | Control | 1,802/37,270 (4.8) | 253,492 | 7.1 | 1 |  | 1 |  |
|  | ≥ 200 to < 240 mg/dL (n = 56,215) | | | | | | | | |
|  |  | Osteoporosis | 2,064/28,105 (7.3) | 203,702 | 10.1 | 1.60 (1.49-1.72) | <0.001* | 1.29 (1.20-1.38) | <0.001* |
|  |  | Control | 1,259/28,110 (4.5) | 199,657 | 6.3 | 1 |  | 1 |  |
|  | ≥ 240 mg/dL (n = 26,866) | | | | | | | | |
|  |  | Osteoporosis | 1,061/13,252 (8.0) | 97,667 | 10.9 | 1.52 (1.38-1.68) | <0.001* | 1.18 (1.07-1.30) | 0.001* |
|  |  | Control | 700/13,614 (5.1) | 98,614 | 7.1 | 1 |  | 1 |  |
| Blood pressure | | | | | | | | | |
|  | SBP < 140 mmHg and DBP < 90 mmHg (n = 111,520) | | | | | | | | |
|  |  | Osteoporosis | 3,647/56,245 (6.5) | 395,102 | 9.2 | 1.63 (1.54-1.72) | <0.001* | 1.31 (1.24-1.39) | <0.001* |
|  |  | Control | 2,095/55,275 (3.8) | 373,351 | 5.6 | 1 |  | 1 |  |
|  | SBP ≥ 140 mmHg or DBP ≥ 90 mmHg (n = 46,468) | | | | | | | | |
|  |  | Osteoporosis | 2,209/22,749 (9.7) | 170,518 | 13.0 | 1.40 (1.31-1.49) | <0.001* | 1.21 (1.13-1.29) | <0.001* |
|  |  | Control | 1,666/23,719 (7.0) | 178,412 | 9.3 | 1 |  | 1 |  |
| Fasting blood glucose | | | | | | | | | |
|  | < 100 mg/dL (n = 105,724) | | | | | | | | |
|  |  | Osteoporosis | 3,653/53,889 (6.8) | 394,788 | 9.3 | 1.60 (1.52-1.69) | <0.001* | 1.30 (1.23-1.38) | <0.001* |
|  |  | Control | 2,120/51,835 (4.1) | 368,729 | 5.7 | 1 |  | 1 |  |
|  | ≥ 100 mg/dL (n = 52,264) | | | | | | | | |
|  |  | Osteoporosis | 2,203/25,105 (8.8) | 170,832 | 12.9 | 1.44 (1.35-1.54) | <0.001* | 1.22 (1.14-1.30) | <0.001* |
|  |  | Control | 1,641/27,159 (6.0) | 183,034 | 9.0 | 1 |  | 1 |  |

Abbreviations: AD, Alzheimer’s disease; CCI, Charlson comorbidity index; DBP, diastolic blood pressure; SBP, systolic blood pressure

* Cox proportional hazard model, Significance at *P* < 0.05 with Bonferroni correction

† Adjusted for age, sex, income, region of residence, total cholesterol, SBP, DBP, fasting blood glucose, obesity, smoking, alcohol consumption, CCI score, and Parkinson’s disease

**Supplementary Table 5**  Hazard ratio (95% confidence interval) for Parkinson’s disease in the osteoporosis and control groups with subgroup analyses according to age and sex

| Characteristics | | Hazard ratios for Parkinson’s disease | | | | | | | | | |
| --- | --- | --- | --- | --- | --- | --- | --- | --- | --- | --- | --- |
|  |  | Crude | *P*-value | Model 1† | *P*-value | Model 2‡ | *P*-value | Model 3§ | *P*-value | Model 4‖ | *P*-value |
| Total participants (n = 157,988) | |  | |  |  |  |  |  |  |  |  |
|  | Osteoporosis | 1.72 (1.57-1.87) | <0.001* | 1.53 (1.40-1.67) | <0.001* | 1.53 (1.40-1.67) | <0.001* | 1.56 (1.43-1.70) | <0.001* | 1.49 (1.36-1.63) | <0.001* |
|  | Control | 1 |  | 1 |  | 1 |  | 1 |  | 1 |  |
| Age < 60 years old (n = 67,714) | |  | |  |  |  |  |  |  |  |  |
|  | Osteoporosis | 1.50 (1.17-1.94) | 0.002* | 1.65 (1.27-2.13) | <0.001* | 1.64 (1.26-2.12) | <0.001* | 1.65 (1.27-2.14) | <0.001* | 1.52 (1.17-1.98) | 0.002* |
|  | Control | 1 |  | 1 |  | 1 |  | 1 |  | 1 |  |
| Age ≥ 60 years old (n = 90,274) | |  | |  |  |  |  |  |  |  |  |
|  | Osteoporosis | 1.41 (1.28-1.55) | <0.001* | 1.42 (1.30-1.56) | <0.001* | 1.42 (1.29-1.56) | <0.001* | 1.45 (1.32-1.59) | <0.001* | 1.40 (1.27-1.53) | <0.001* |
|  | Control | 1 |  | 1 |  | 1 |  | 1 |  | 1 |  |
| Males (n = 21,213) | |  | |  |  |  |  |  |  |  |  |
|  | Osteoporosis | 1.31 (1.07-1.60) | 0.008* | 1.61 (1.31-1.97) | <0.001* | 1.60 (1.31-1.97) | <0.001* | 1.62 (1.32-1.99) | <0.001* | 1.49 (1.21-1.83) | <0.001* |
|  | Control | 1 |  | 1 |  | 1 |  | 1 |  | 1 |  |
| Females (n = 136,775) | |  | |  |  |  |  |  |  |  |  |
|  | Osteoporosis | 1.87 (1.69-2.06) | <0.001* | 1.46 (1.32-1.61) | <0.001* | 1.46 (1.32-1.61) | <0.001* | 1.49 (1.35-1.64) | <0.001* | 1.46 (1.33-1.62) | <0.001* |
|  | Control | 1 |  | 1 |  | 1 |  | 1 |  | 1 |  |

Abbreviations: CCI, Charlson comorbidity index; DBP, diastolic blood pressure; SBP, systolic blood pressure

* Cox proportional hazard model, Significance at *P* < 0.05 with Bonferroni correction

† Model 1 was adjusted for age, sex, income, region, and obesity

‡ Model 2 was adjusted for model 1 plus smoking, and alcohol consumption

§ Model 3 was adjusted for model 2 plus total cholesterol, SBP, DBP, and fasting blood glucose

‖ Model 4 was adjusted for model 3 plus CCI score, and Alzheimer's disease

**Supplementary Table** **6** Hazard ratio (95% confidence interval) for Parkinson’s disease in the osteoporosis and control groups with subgroup analyses according to income, region of residence, obesity, smoking, alcohol consumption, total cholesterol, blood pressure, and fasting blood glucose

| Characteristics | | | No. of PD/ No. of participants | Follow-up duration, person-years | Incidence rate,  per 1,000  person-years | Hazard ratios for PD | | | |
| --- | --- | --- | --- | --- | --- | --- | --- | --- | --- |
|  |  |  |  |  |  | Crude | *P*-value | Adjusted† | *P*-value |
| Income | | | | | | | | | |
|  | Low income (n = 77,752) | | | | | | | | |
|  |  | Osteoporosis | 679/38,723 (1.8) | 282,620 | 2.4 | 1.83 (1.61-2.07) | <0.001* | 1.58 (1.39-1.80) | <0.001* |
|  |  | Control | 363/39,029 (0.9) | 275,776 | 1.3 | 1 |  | 1 |  |
|  | High income (n = 80,236) | | | | | | | | |
|  |  | Osteoporosis | 718/40,271 (1.8) | 294,946 | 2.4 | 1.62 (1.44-1.83) | <0.001* | 1.41 (1.24-1.59) | <0.001* |
|  |  | Control | 427/39,965 (1.1) | 284,301 | 1.5 | 1 |  | 1 |  |
| Region of residence | | | | | | | | | |
|  | Urban (n = 60,783) | | | | | | | | |
|  |  | Osteoporosis | 478/30,828 (1.6) | 228,966 | 2.1 | 1.71 (1.47-1.98) | <0.001* | 1.56 (1.34-1.81) | <0.001* |
|  |  | Control | 268/29,955 (0.9) | 218,857 | 1.2 | 1 |  | 1 |  |
|  | Rural (n = 97,205) | | | | | | | | |
|  |  | Osteoporosis | 919/48,166 (1.9) | 348,600 | 2.6 | 1.72 (1.54-1.92) | <0.001* | 1.45 (1.30-1.62) | <0.001* |
|  |  | Control | 522/49,039 (1.1) | 341,220 | 1.5 | 1 |  | 1 |  |
| Obesity | | | | | | | | | |
|  | Underweight (n = 4,443) | | | | | | | | |
|  |  | Osteoporosis | 49/2,573 (1.9) | 16,933 | 2.9 | 1.34 (0.83-2.17) | 0.232 | 1.27 (0.78-2.07) | 0.346 |
|  |  | Control | 25/1,870 (1.3) | 11,542 | 2.2 | 1 |  | 1 |  |
|  | Normal weight (n = 56,634) | | | | | | | | |
|  |  | Osteoporosis | 498/29,444 (1.7) | 212,228 | 2.3 | 1.76 (1.51-2.05) | <0.001* | 1.58 (1.36-1.85) | <0.001* |
|  |  | Control | 246/27,190 (0.9) | 183,670 | 1.3 | 1 |  | 1 |  |
|  | Overweight (n = 41,399) | | | | | | | | |
|  |  | Osteoporosis | 354/20,665 (1.7) | 152,365 | 2.3 | 1.83 (1.54-2.19) | <0.001* | 1.58 (1.32-1.89) | <0.001* |
|  |  | Control | 187/20,734 (0.9) | 147,656 | 1.3 | 1 |  | 1 |  |
|  | Obese (n = 55,512) | | | | | | | | |
|  |  | Osteoporosis | 496/26,312 (1.9) | 196,040 | 2.5 | 1.66 (1.44-1.90) | <0.001* | 1.37 (1.19-1.58) | <0.001* |
|  |  | Control | 332/29,200 (1.1) | 217,209 | 1.5 | 1 |  | 1 |  |
| Smoking | | | | | | | | | |
|  | Nonsmoker (n = 143,721) | | | | | | | | |
|  |  | Osteoporosis | 1,271/72,716 (1.8) | 540,033 | 2.4 | 1.77 (1.61-1.94) | <0.001* | 1.48 (1.34-1.62) | <0.001* |
|  |  | Control | 689/71,005 (1.0) | 516,854 | 1.3 | 1 |  | 1 |  |
|  | Past or current smoker (n = 14,267) | | | | | | | | |
|  |  | Osteoporosis | 126/6,278 (2.0) | 37,533 | 3.4 | 1.43 (1.10-1.86) | 0.007* | 1.50 (1.15-1.96) | 0.003* |
|  |  | Control | 101/7,989 (1.3) | 43,223 | 2.3 | 1 |  | 1 |  |
| Alcohol consumption | | | | | | | | | |
|  | < 1 time a week (n = 137,764) | | | | | | | | |
|  |  | Osteoporosis | 1,286/70,097 (1.8) | 529,499 | 2.4 | 1.72 (1.57-1.89) | <0.001* | 1.48 (1.35-1.63) | <0.001* |
|  |  | Control | 709/67,667 (1.1) | 502,143 | 1.4 | 1 |  | 1 |  |
|  | ≥ 1 time a week (n = 20,224) | | | | | | | | |
|  |  | Osteoporosis | 111/8,897 (1.3) | 48,067 | 2.3 | 1.65 (1.24-2.20) | 0.001* | 1.58 (1.18-2.11) | 0.002* |
|  |  | Control | 81/11,327 (0.7) | 57,934 | 1.4 | 1 |  | 1 |  |
| Total cholesterol | | | | | | | | | |
|  | < 200 mg/dL (n = 74,907) | | | | | | | | |
|  |  | Osteoporosis | 672/37,637 (1.8) | 269,473 | 2.5 | 1.83 (1.61-2.08) | <0.001* | 1.66 (1.46-1.90) | <0.001* |
|  |  | Control | 352/37,270 (0.9) | 257,425 | 1.4 | 1 |  | 1 |  |
|  | ≥ 200 to < 240 mg/dL (n = 56,215) | | | | | | | | |
|  |  | Osteoporosis | 477/28,105 (1.7) | 208,134 | 2.3 | 1.62 (1.40-1.87) | <0.001* | 1.36 (1.17-1.57) | <0.001* |
|  |  | Control | 287/28,110 (1.0) | 202,448 | 1.4 | 1 |  | 1 |  |
|  | ≥ 240 mg/dL (n = 26,866) | | | | | | | | |
|  |  | Osteoporosis | 248/13,252 (1.9) | 99,959 | 2.5 | 1.64 (1.34-2.01) | <0.001* | 1.35 (1.10-1.66) | 0.004* |
|  |  | Control | 151/13,614 (1.1) | 100,204 | 1.5 | 1 |  | 1 |  |
| Blood pressure | | | | | | | | | |
|  | SBP < 140 mmHg and DBP < 90 mmHg (n = 111,520) | | | | | | | | |
|  |  | Osteoporosis | 873/56,245 (1.6) | 402,579 | 2.2 | 1.83 (1.63-2.05) | <0.001* | 1.54 (1.37-1.73) | <0.001* |
|  |  | Control | 447/55,275 (0.8) | 377,866 | 1.2 | 1 |  | 1 |  |
|  | SBP ≥ 140 mmHg or DBP ≥ 90 mmHg (n = 46,468) | | | | | | | | |
|  |  | Osteoporosis | 524/22,749 (2.3) | 174,987 | 3.0 | 1.59 (1.39-1.82) | <0.001* | 1.42 (1.24-1.63) | <0.001* |
|  |  | Control | 343/23,719 (1.5) | 182,211 | 1.9 | 1 |  | 1 |  |
| Fasting blood glucose | | | | | | | | | |
|  | < 100 mg/dL (n = 105,724) | | | | | | | | |
|  |  | Osteoporosis | 850/53,889 (1.6) | 402,500 | 2.1 | 1.77 (1.58-1.99) | <0.001* | 1.49 (1.32-1.67) | <0.001* |
|  |  | Control | 445/51,835 (0.9) | 373,715 | 1.2 | 1 |  | 1 |  |
|  | ≥ 100 mg/dL (n = 52,264) | | | | | | | | |
|  |  | Osteoporosis | 547/25,105 (2.2) | 175,066 | 3.1 | 1.69 (1.48-1.93) | <0.001* | 1.49 (1.30-1.71) | <0.001* |
|  |  | Control | 345/27,159 (1.3) | 186,362 | 1.9 | 1 |  | 1 |  |

Abbreviations: Parkinson’s disease, PD; CCI, Charlson comorbidity index; DBP, diastolic blood pressure; SBP, systolic blood pressure

* Cox proportional hazard model, Significance at *P* < 0.05 with Bonferroni correction

† Adjusted for age, sex, income, region of residence, total cholesterol, SBP, DBP, fasting blood glucose, obesity, smoking, alcohol consumption, CCI score, and Alzheimer's disease

**Supplementary Table** **7** The number and proportion of incident Alzheimer's disease occurred in osteoporosis and control according to age distribution

| Age (years) | No. of Alzheimer's disease (%)/ No. of osteoporosis (%) | No. of Alzheimer's disease (%)/ No. of control (%) |
| --- | --- | --- |
| 40-44 | 5/892 (0.6) | 0/892 (0.0) |
| 45-49 | 12/4,422 (0.3) | 15/4,422 (0.3) |
| 50-54 | 63/10,002 (0.6) | 51/10,002 (0.5) |
| 55-59 | 213/11,511 (1.9) | 191/25,571 (0.8) |
| 60-64 | 619/13,458 (4.6) | 467/15,268 (3.1) |
| 65-69 | 1,410/16,736 (8.4) | 738/7,815 (9.4) |
| 70-74 | 1,755/12,761 (13.8) | 879/6,556 (13.4) |
| 75-79 | 1,232/6,674 (18.5) | 714/3,980 (17.9) |
| 80-84 | 476/2,207 (21.6) | 406/2,232 (18.2) |
| 85+ | 71/331 (21.5) | 300/2,256 (13.3) |

**Supplementary Table** **8** The number and proportion of incident Parkinson’s disease occurred in osteoporosis and control according to age distribution

| Age (years) | No. of Parkinson's disease (%)/ No. of osteoporosis (%) | No. of Parkinson's disease (%)/ No. of control (%) |
| --- | --- | --- |
| 40-44 | 2/892 (0.2) | 1/892 (0.1) |
| 45-49 | 11/4,422 (0.3) | 8/4,422 (0.2) |
| 50-54 | 42/10,002 (0.4) | 20/10,002 (0.2) |
| 55-59 | 74/11,511 (0.6) | 81/25,571 (0.3) |
| 60-64 | 212/13,458 (1.6) | 157/15,268 (1.0) |
| 65-69 | 397/16,736 (2.4) | 167/7,815 (2.1) |
| 70-74 | 373/12,761 (2.9) | 181/6,556 (2.8) |
| 75-79 | 222/6,674 (3.3) | 95/3,980 (2.4) |
| 80-84 | 62/2,207 (2.8) | 44/2,232 (2.0) |
| 85+ | 2/331 (0.6) | 36/2,256 (1.6) |

**Supplementary Table** **9** The number and proportion of incident Alzheimer's disease in osteoporosis and control according to sex

| Sex | No. of Alzheimer's disease (%)/ No. of osteoporosis (%) | No. of Alzheimer's disease (%)/ No. of control (%) |
| --- | --- | --- |
| Males | 712/9,138 (7.8) | 953/12,075 (7.9) |
| Females | 5,144/69,856 (7.4) | 2,808/66,919 (4.2) |

**Supplementary Table** **10** The number and proportion of incident Parkinson’s disease occurred in osteoporosis and control according to sex

| Sex | No. of Parkinson's disease (%)/ No. of osteoporosis (%) | No. of Parkinson's disease (%)/ No. of control (%) |
| --- | --- | --- |
| Males | 210/9,138 (2.3) | 184/12,075 (1.5) |
| Females | 1,187/69,856 (1.7) | 606/66,919 (0.9) |

**Supplementary Figure 1.** The proportional hazard assumptions were checked using log-minus-log plots for Alzheimer’s disease and Parkinson’s disease, and no violations of these assumptions were found.


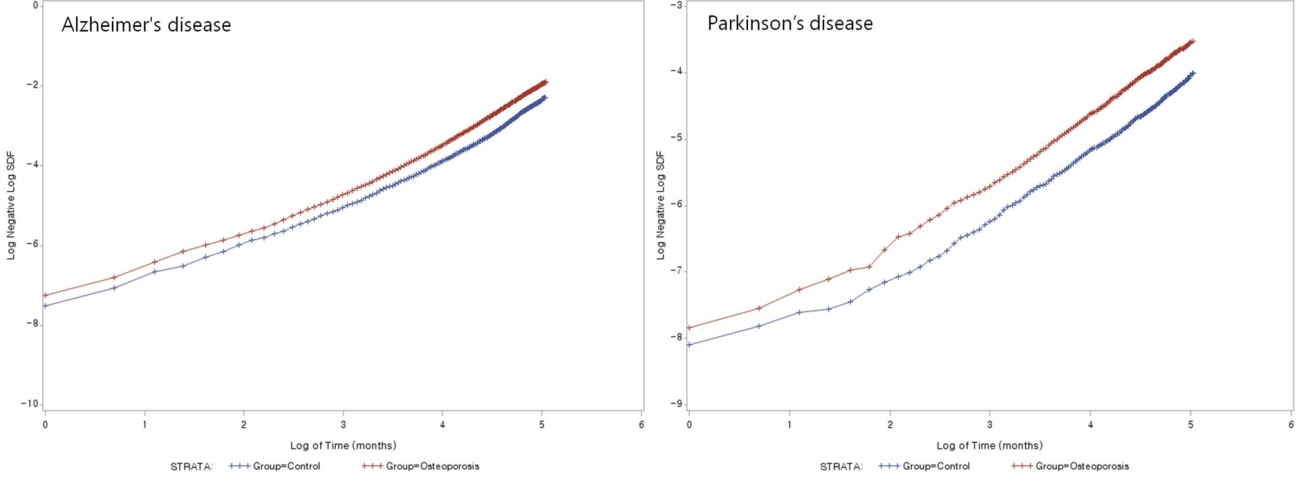

Supplement: Supplementary file 1 [file Data_Sheet_1.docx]
